# Effects of Relaxed Lockdown on Pediatric ER Visits during SARS-CoV-2 Pandemic in Italy

**DOI:** 10.3390/ijerph18189547

**Published:** 2021-09-10

**Authors:** Luigi Matera, Raffaella Nenna, Francesca Ardenti Morini, Giuseppe Banderali, Mauro Calvani, Matteo Calvi, Giorgio Cozzi, Raffaele Falsaperla, Roberto Guidi, Ahmad Kantar, Marcello Lanari, Riccardo Lubrano, Beatrice Messini, Antonio Augusto Niccoli, Vincenzo Tipo, Fabio Midulla, on behalf of the CICERO Group

**Affiliations:** 1 Department of Maternal, Infantile and Urological Sciences, Sapienza University of Rome, 00161 Rome, Italy; luigi.matera@uniroma1.it (L.M.); raffaella.nenna@uniroma1.it (R.N.); 2 Pediatric Unit, Sant’Eugenio Hospital, 00144 Rome, Italy; francesca.ardentimorini@aslroma2.it; 3 Department of Clinical Paediatrics, San Paolo Hospital, University of Milan, 20142 Milan, Italy; giuseppe.banderali@unimi.it; 4 Operative Unit of Pediatrics, San Camillo-Forlanini Hospital, 00151 Rome, Italy; maurocalvani58@gmail.com; 5 Pediatric Emergency Department, Papa Giovanni XXIII Hospital, 24127 Bergamo, Italy; mcalvi@asst-pg23.it; 6 Institute for Maternal and Child Health Burlo Garofalo, 34137 Trieste, Italy; giorgio.cozzi@burlo.trieste.it; 7 General Pediatrics and Pediatric Acute and Emergency Unit, Vittorio Emanuele University Hospital, 95121 Catania, Italy; raffaelefalsaperla@hotmail.com; 8 Department of Pediatric Emergency, G. Salesi Hospital, 60123 Ancona, Italy; roberto.guidi@ospedaliriuniti.marche.it; 9 Pediatric Unit, Istituti Ospedalieri Bergamaschi, Gruppo Ospedaliero San Donato, 24059 Ponte San Pietro, Italy; kantar@centropediatricotosse.com; 10 Pediatric Emergency Unit, Scientific Institute for Research and Healthcare, Sant’Orsola Hospital, 40138 Bologna, Italy; marcello.lanari@unibo.it; 11 Pediatric Unit, Department of Maternal and Child Health, Santa Maria Goretti Hospital, Sapienza University of Rome, 04100 Latina, Italy; riccardo.lubrano@uniroma1.it; 12 Pediatric Unit, San Giovanni Battista Hospital, 06034 Foligno, Italy; bmessini@alice.it; 13 Pediatric Unit, Fabrizio Spaziani Hospital, 03100 Frosinone, Italy; dr.aniccoli@gmail.com; 14 Pediatric Emergency Department, Santobono-Pausilipon Hospital, 06049 Napoli, Italy; enzotipo@libero.it

**Keywords:** air communicable infections, emergency rooms, social activities reopening, lockdown measures, pediatric, pandemic, SARS-CoV-2, COVID-19

## Abstract

Previously, we demonstrated an 81% reduction in pediatric Emergency Room (ER) visits in Italy during the strict lockdown due to the SARS-CoV-2 pandemic. Since May 2020, lockdown measures were relaxed until 6 November 2020, when a strict lockdown was patchily reintroduced. Our aim was to evaluate the impact of the relaxed lockdown on pediatric ER visits in Italy. We performed a retrospective multicenter study involving 14 Italian pediatric ERs. We compared total ER visits from 24 September 2020 to 6 November 2020 with those during the corresponding timeframe in 2019. We evaluated 17 ER specific diagnoses grouped in air communicable and non-air communicable diseases. We recognized four different triage categories: white, green, yellow and red. In 2020 total ER visits were reduced by 51% compared to 2019 (16,088 vs. 32,568, respectively). The decrease in air communicable diseases was significantly higher if compared to non-air communicable diseases (−64% vs. −42%, respectively). ER visits in each triage category decreased in 2020 compared to 2019, but in percentage, white and red codes remained stable, while yellow codes slightly increased and green codes slightly decreased. Our results suggest that preventive measures drastically reduced the circulation of air communicable diseases even during the reopening of social activities but to a lesser extent with regard to the strict lockdown period (March–May 2020).

## 1. Introduction

The first cases of Severe Acute Respiratory Syndrome Coronavirus 2 (SARS-CoV-2) were described in China in December 2019 and the WHO declared a pandemic on 11 March 2020 [1]. The first Italian case was described in February 2020. Since that date, a total of 4,515,967 cases and 128,362 deaths (2.8%) were reported in Italy, with a median age of 46 years [2]. Until 25 August 2021, 711,898 (15.9%) pediatric cases were reported in Italy, with 31 fatalities [2].

In order to face this pandemic spread, the Italian Prime Minister declared a strict lockdown on 9 March 2020 [3]. Bans of mass gatherings, social distancing and the obligation of wearing face masks starting at six years of age were introduced. Moreover, simple hygiene measures, such as hand washing, were implemented. Mass closures followed, including schools, factories, pubs, shopping centers and restaurants. Only supermarkets remained open, providing daily necessities. Workers were encouraged to use smart-working. Breaking the quarantine was allowed only in case of extreme necessities. These preventive measures were operative until 3 May 2020, when they were relaxed [4]: factories, shopping malls, pubs and restaurants were reopened, while schools remained closed. People were allowed to leave their home and take summer holidays. From 14 September 2020 to 24 September 2020, schools were reopened in Italy [5], implementing the environmental and surveillance measures in order to face the in-school transmission. The obligation to wear face masks, handwashing and bans of mass gatherings were highlighted as cornerstones in order to prevent the spread of SARS-CoV-2 in children and, consequently, from children to adults [5].

In a previous Italian epidemiologic study including 15 hospitals over eight Italian regions, we showed an 81% decrease in pediatric Emergency Room (ER) visits during March–May 2020 compared to the corresponding timeframes in 2019, with the largest drop affecting air communicable diseases [6]. The drastic preventive measures applied during the strict lockdown can explain these results [6]. It would be useful to know what happened when more permissive measures were applied.

In this multicentric study, our aim was the evaluation of how the SARS-CoV-2 preventive strategies, such as social distancing, face masks and hygiene measures, affected pediatric ER visits when lockdown measures were relaxed, allowing the reopening of social activities in Italy. We compared ER visit rates, specific ER diagnoses and triage categories from 24 September 2020 to 6 November 2020 with the corresponding timeframe in 2019.

## 2. Materials and Methods

**Figure 1:**
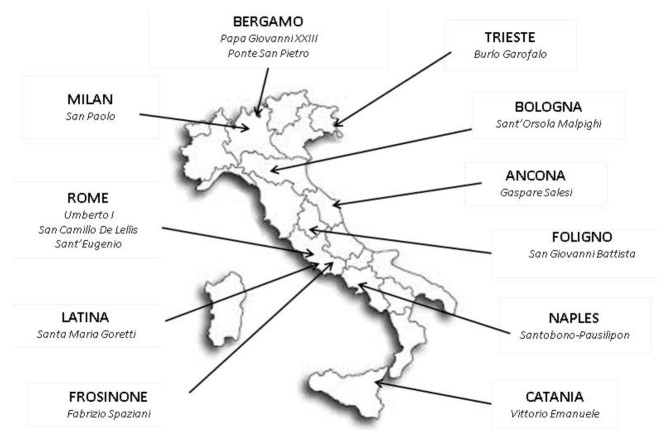
Pediatric Emergency Rooms (ERs) included in the study.

We performed a retrospective multicentric study involving 14 Italian pediatric ERs, from northern to southern Italy (Figure 1). We randomly selected 14 public hospitals throughout Italy, thus providing a representative sample of the entire Italian population. We collected data from ERs located in Ancona, Bergamo, Bologna, Catania, Foligno, Frosinone, Latina, Milan, Naples, Rome and Trieste, which represented about 10% of the total pediatric ERs visits per year in Italy [6].

We compared two corresponding timeframes in 2019 and 2020, starting from 24 September. In Italy, schools were opened patchily in the different regions, because each region itself decided the dates in which they should be reopened. The reopenings started from the first days of September and continued until 24 September. In order to avoid confounding factors, we decided to start our analysis on 24 September 2020, when schools were opened throughout the national territory. For the same reason, we decided to stop our analysis on 6 November 2020 because, since that date, a new legislation was adopted in Italy, establishing a further differentiation between Italian regions based on their pandemic critical features. The classification assigned different colors to each region, indicating its SARS-CoV-2 pandemic background, from the least to the most severe: white, yellow, orange and red. These different features determined different strict lockdown measures, such as different curfew times, obligation to wear face masks outdoors, the opening of restaurants and shopping centers and different social activities proceedings, from their complete closure to their opening.

Firstly, we compared these two reference periods in terms of total visits in the 14 pediatric ERs participating in the study. Subsequently, consistent with our previous study [6], we evaluated 17 ER specific diagnoses, based on the primary discharge diagnoses ruled out in ER according to the ICD9-CM codes. These diagnoses were grouped into air communicable and non-air communicable diseases [6]. In the group of air communicable diseases, we included upper and lower respiratory infections, gastroenterological infections and exanthematous diseases, and we assumed that these conditions were related to airborne transmission. Non-air communicable diseases included accidents, cardiovascular, dermatological diseases, endocrinological disorders, fever and surgical pathologies, as well as hematological, nephrological, neurological, neuropsychiatric, oncological, ophthalmological and rheumatological diseases, because we assumed that these conditions were not related to airborne transmission. Fever was included in this second category because, if it occurs without any other signs or symptoms, it is more likely related to non-airborne infections, such as urinary tract infections. In order to better understand our analysis, we also investigated the trend in respiratory diseases alone compared to the other 16 specific diagnoses evaluated in this study.

Similarly, we recognized four different triage categories, from the least to the most severe: white, green, yellow, and red, which were standardized combining the orange and blue codes as yellow codes, as previously described [6]. In fact, since 2019 some regions, including Lazio, adopted new triage colors and defined five new triage categories with different priority codes: red (immediate access), orange (access in 15 min), blue (access in 60 min), green (access in 120 min) and white (access in 240 min). To standardize our data, we combined the orange and blue codes as yellow codes.

We performed our statistical analysis using IBM SPSS Statistics v.25 (IBM Corp. Released 2017. IBM SPSS Statistics for Windows, Version 25.0. Armonk, NY: IBM Corp.). We compared the ER visits rates, ER specific diagnoses in terms of air communicable diseases and non-air communicable diseases and triage categories in 2019 vs. 2020 by chi-square tests. A *p*-value < 0.05 was considered statistically significant.

## 3. Results

We collected data from 14 Italian pediatric ERs, from 24 September to 6 November, 2019 and 2020: Umberto I hospital, Rome (2901 vs. 1338); San Camillo de Lellis hospital, Rome (1587 vs. 711); Sant’Eugenio hospital, Rome (591 vs. 222); Santa Maria Goretti hospital, Latina (643 vs. 283); Vittorio Emanuele hospital, Catania (874 vs. 374); San Paolo hospital, Milan (1489 vs. 720); Burlo Garofalo hospital, Trieste (3041 vs. 1894); Ponte San Pietro hospital, Bergamo (1297 vs. 481); Papa Giovanni XXIII hospital, Bergamo (1952 vs. 864); Santobono-Pausilipon hospital, Naples (11,062 vs. 5032); Sant’Orsola hospital, Bologna (2709 vs. 1941); Fabrizio Spaziani hospital, Frosinone (1323 vs. 527); San Giovanni Battista hospital, Foligno (625 vs. 253); and Gaspare Salesi hospital, Ancona (2474 vs. 1448).

Comparing total ER visits from 24 September to 6 November 2020, with those from 24 September to 6 November 2019, we observed a reduction rate of 51% (32,568 vs. 16,088 visits, respectively) (*p* < 0.001) (Table 1).

**Figure 2:**
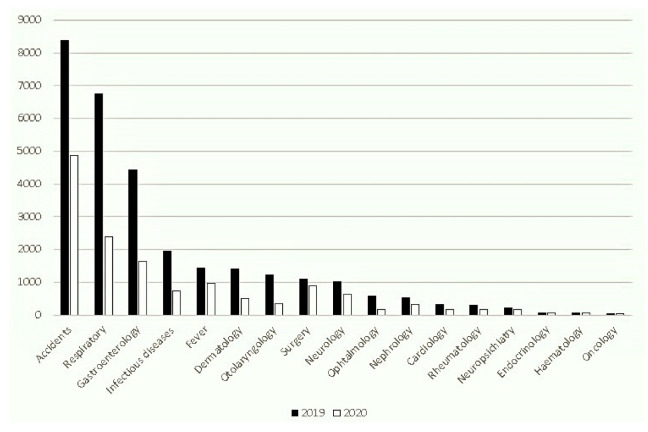
Pediatric ER diagnoses in September–November, 2019 and 2020.

We encountered a significant reduction in visits for all 17 clinical categories evaluated in the 2020 period compared to 2019 (Figure 2).

**Figure 3:**
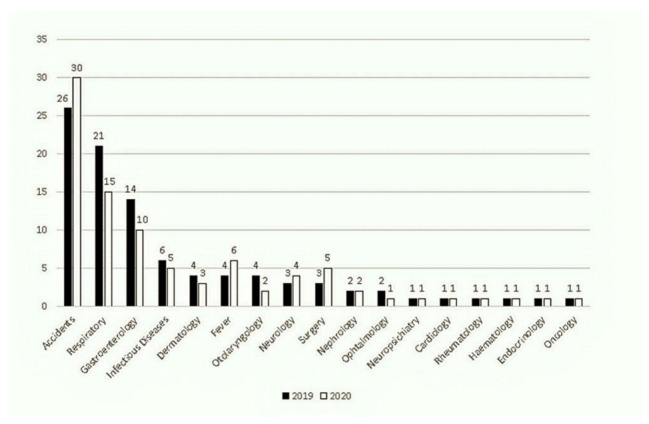
Proportion of pediatric ER diagnoses in September–November, 2019 and 2020.

Nevertheless, for some categories, such as accidents, we observed a relative increase in percentage when compared to the total number of visits per reference period (Figure 3).

The decrease in air communicable diseases (13,134 vs. 4754: −63.8%) was significantly higher compared to non-air communicable diseases (19,434 vs. 11,334: −41.7%) (*p* < 0.001). Analyzing respiratory diseases alone, we found a statistical significantly decrease in respiratory diseases (6743 vs. 2393, −4%) compared to the total amount of the others 16 specific diagnoses evaluated in this study (25,296 vs. 11,667, +4%) (*p* < 0.001).

**Figure 4:**
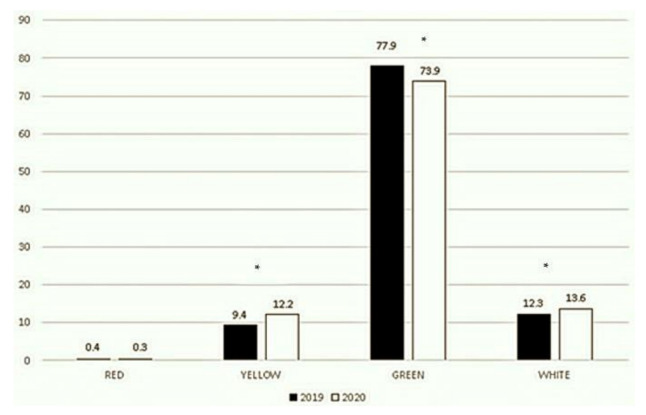
Triage categories in September–November, 2019 and 2020. This figure shows the breakdown of 100% of ER visits into 4 codes. * *p* < 0.001.

ER visits in each triage category decreased in 2020. Our data showed 118 vs. 43 red codes, 3083 vs. 1954 yellow codes, 25,369 vs. 11,898 green codes, and 3998 vs. 2189 white codes in 2019 vs. 2020, respectively. By contrast, considering the proportion of each category over the total number of visits per reference period, the red codes remained almost similar (0.4% vs. 0.3%, *p* = 0.85), while the yellow codes increased (9.4% vs. 12.2%, *p* < 0.001), green codes decreased (77.9% vs. 73.9%, *p* < 0.001), and white codes slightly increased (12.3% vs. 13.6%, *p* < 0.001) (Figure 4).

## 4. Discussion

In our study, evaluating 14 Italian hospitals that are a representative sample of the whole Italian population, the most important result is that total pediatric ER visits dropped by 51% from 24 September to 6 November 2020, compared to the corresponding timeframe in 2019. After demonstrating a significant reduction of ER visits during the strict lockdown (9 March to 3 May 2020) in our previous manuscript [6], we rolled out this study in order to assess the effect of relaxed preventing restrictions, such as social activities reopening. We found a significantly lower drop in ER visits in September–November 2020 compared to March–May 2020 (−51% vs. −81%, respectively). Our results are supported by other studies that have demonstrated a drastic fall in ER visits both in children and in adults [6,7,8,9,10,11,12,13]. This drop can be explained by the preventive measures adopted in Italy, such as social-distancing measures, the use of face masks, hand washing and bans of mass gatherings, which may have contributed to the reduction of the spread of both SARS-CoV-2 and other acute communicable diseases, the most common ER presentations in children along with accidents [6,7]. Moreover, in the present study, we confirmed the reduction in all 17 clinical categories, regarding both air communicable and non-air communicable diseases, but similarly to above, the reduction of air communicable diseases was significantly lower in September–November 2020 when compared to March–May 2020 (−64% vs. −88.5%, respectively). We can speculate that the reopening of social activities, with the mitigation of lockdown measures in May 2020 and in particular the reopening of schools in September 2020, at least partially allowed the circulation of air communicable diseases. Our speculations are supported by other studies that have demonstrated an increase in air communicable diseases’ diffusion after the mitigation of lockdown, both in children and in adults [14,15,16]. By contrast, during the strict lockdown, air communicable diseases’ diffusion was really reduced [6,16,17,18,19,20], leading to a drastic drop in ER visits [9,10,11,13], as we have already demonstrated in our previous study [6]. These results highlight the importance of preventive measures in order to face acute communicable diseases’ diffusion, in particular in pediatric age, because several studies have shown that children are at higher risk of infections [17,18,21] and are pivotal in air communicable diseases’ transmission [22]. Our preliminary data, analyzing a one year surveillance period from March 2020 to February 2021, showed that RSV and other viruses (including influenza virus A and B, human coronavirus OC43, 229E, NL-63 and HUK1, adenovirus, parainfluenza virus 1-3, human bocavirus and human metapneumovirus) almost disappeared, while human rhinovirus (hRV) was the only detected virus. In fact, evaluating 86 hospitalized children, we found hRV in 41, RSV in 4 and other viruses in 1. Interestingly, hRV did not show a peak in spring 2020 as the other epidemic seasons, but in autumn–winter 2020, it was the one detected in hospitalized children, showing a spread trend comparable to other epidemic seasons. Thus, the relatively low efficacy of surgical masks, along with hRV features and the reduced social distancing in social activities, allowed the circulation of the virus.

Every year in Italy, approximately 3 million children are admitted to ERs [23]. Approximately 0.5–1% are classified as red codes and 10–12% as yellow codes, meaning that approximately 20,000–30,000 children seek medical advice for life-threatening clinical conditions and about 300,000 children present with serious conditions each year. Nevertheless, approximately 70–80% of ER visits are categorized as green codes. This considerable number of non-urgent patients with acute clinical conditions, which could be used to treat with in an outpatient setting, has necessarily led to the well-known and harmful overcrowding of ER departments [23]. Evaluating triage categories, we demonstrated a consistent reduction in all ER visits. This reduction goes along with the reduction in the spread of air communicable diseases [6,7] and in outdoor accidents [6], which represent the most frequent clinical presentations in pediatric ERs. Moreover, we cannot rule out that parents’ fear of SARS-CoV-2 [6,8,9,24,25] and the strict measures adopted in Italy [6,8,26] have contributed to this reduction. Another interesting result that came out comparing our two studies was that in both reference periods, yellow codes increased, and green codes decreased compared to their corresponding timeframes in 2019 but, in September–November 2020, yellow codes increased, and green codes decreased to a lesser extent than March–May 2020, when lockdown measures were more stringent (yellow codes: +11.1% vs. +2.8%; green codes: −10.8% vs. −4%, respectively) [6]. These results may highlight that a lesser sense of fear concerning SARS-CoV-2 [27] and more organized primary cares have limited the number of patients that delayed their ER visits, in contrast to what happened during the first wave of pandemic [28,29,30,31,32]. In particular, a more intense sense of fear was demonstrated in people living in high-death-rate countries with strict lockdown measures [26]. Thus, the mitigation of lockdown measures could have led people to have a lesser sense of fear in respect to SARS-CoV-2, avoiding delayed ER visits.

An interesting result came out analyzing the trend in ER visits for accidents considering the strict and the relaxed lockdown period. Accidents, together with air communicable diseases, typically represent the main clinical presentation in pediatric ERs [6,7]. In our previous work, comparing the strict lockdown period (March–May 2020) with the same period in 2019, we demonstrated 11148 ER visits for accidents in 2019 compared to 3380 in 2020 (−70%) [6]. Our results were superimposable on other studies that reported reductions in injuries in pediatric age in UK, Germany, Belgium, France, Italy, USA, South Africa and Singapore [13,17,33,34,35,36,37]. Along this hypothesis, several studies demonstrated a drastic drop in pediatric ER visits for accidents during the lockdown period [6,38,39]. This decreasing trend is confirmed also by more recent published works that analyzed different timeframes, in particular until June [40] and August [41] 2020, when strict lockdown measures were patchily reintroduced. In the present work, analyzing ER visits in September–November 2020, we confirm this decreasing trend in accidents even when lockdown measures were relaxed, allowing several social activities reopening, such as schools. In fact, we found 8384 ER visits for accidents in September–November 2019 compared to 4871 in the same timeframe in 2020 (−42%). We can compare accident visits during the strict lockdown and relaxed lockdown period. Interestingly, we found 3380 vs. 4871 accident visits (+31%) [6]. We can speculate that, during the strict lockdown, the increased parental supervision and the school, sport activities and playground closures may have played a pivotal role in the reduction of accidental injuries [42]. However, when strict measures were relaxed, the increase in opportunities for trauma led to an increase in accidents visits. On the other hand, we cannot forget that the home environment remains a frequent place for accidents in children [43,44]. In fact, we found a marked increase in the proportion of injuries both during the strict [6] and relaxed lockdown periods.

An important decision that was made during the relaxed lockdown was the reopening of schools. In fact, an important effect of the SARS-CoV-2 pandemic was school closure that affected thousands of children worldwide for about one year. By 26 April 2020, the United Nations Education, Scientific and Cultural Organization (UNESCO) estimated that 1,451,874,449 learners were affected globally. Thus, is school closure beneficial? It is known that the SARS-CoV-2 infection in children is mostly a mild disease [45]. However, we cannot forget that children are susceptible in any case to the infection and then they can spread the virus [46,47]. The mild clinical presentation arises concerns about in-school transmission, as children are seen as super spreaders. Several studies highlighted that child contribute minimally to SARS-CoV-2 growth rates [48,49,50,51,52,53,54]. The opening of schools may be considered safe where there is low SARS-CoV-2 background prevalence. In fact, several data indicate that adults play a crucial role in spreading the virus to their households [55,56] and that in-school SARS-CoV-2 transmission depends on local background prevalence [57,58]. Thus, improving preventing measures (such as decreasing class sizes, organizing different timetables, holding lessons outdoors, physical distancing, hygiene measures, face masks and the obligation to stay at home when sick) are crucial to minimizing the risk of in-class transmission [46,47,59,60]. It is important to remember that social activities closures can lead to potential harmful consequences for the school-aged population and their families, concerning obesity and malnutrition, immunization rates, domestic violence and child abuse [57], delays or precocity in cognitive, physical or social growth, later diagnoses of developmental conditions and overall health inequity [61,62,63,64,65,66]. Thus, school closures should be correctly assessed balancing the risk/benefit ratio, protecting, on the one side, the child health and, on the other side, their crucial growth milestones.

This study has some limitations. Social distancing or other preventive strategies were not directly measured in order to evaluate their contribution in reducing the spread of SARS-CoV-2 and other air communicable infections. We did not investigate the parents’ feelings about keeping their children at home in spite of medical problems due to the fear of the pandemic. We focused the analysis on a period in which lockdown measures were relaxed, and we assumed that these measures, such as social distancing, face masks and hand washing, were followed.

## 5. Conclusions

Our results suggest that preventive measures, such as face masks, social distancing and simple hygiene measures, were associated with a reduction in air communicable diseases’ spread even during the reopening of social activities and schools in particular but to a lesser extent with respect to the lockdown period (March–May 2020).

The slight percentage increase in yellow codes and the slight percentage decrease in green codes in September–November 2020 compared to lockdown period may indicate an improvement in primary cares and, consequently, a reduction of delayed visits at pediatric ERs.

## Figures and Tables

**Table 1:** ER visits registered in September–November, 2019 and 2020.

Hospital	2019	2020		*p*-Value
Umberto I–Rome	2901	1338	−54%	*p* < 0.001
San Camillo de Lellis–Rome	1587	711	−55%	
Sant’Eugenio–Rome	591	222	−52%	
Santa Maria Goretti–Latina	643	283	−56%	
Vittorio Emanuele–Catania	874	374	−57%	
San Paolo–Milan	1489	720	−52%	
Burlo Garofalo–Trieste	3041	1894	−38%	
Ponte San Pietro–Bergamo	1297	481	−63%	
Papa Giovanni XXIII–Bergamo	1952	864	−56%	
Santobono-Pausilipon–Naples	11,062	5032	−54%	
Sant’Orsola–Bologna	2709	1941	−28%	
Fabrizio Spaziani–Frosinone	1323	527	−60%	
San Giovanni Battista–Foligno	625	253	−60%	
Gaspare Salesi–Ancona	2474	1448	−41%	
TOTAL	32,568	16,088	−51%	
